# Plant Small Non-coding RNAs and Their Roles in Biotic Stresses

**DOI:** 10.3389/fpls.2018.01038

**Published:** 2018-07-20

**Authors:** Eleanor J. Brant, Hikmet Budak

**Affiliations:** Cereal Genomics Laboratory, Department of Plant Sciences and Plant Pathology, Montana State University, Bozeman, MT, United States

**Keywords:** miRNA, snRNA, snoRNA, sRNA mobility, plant–pathogen interactions

## Abstract

Non-coding RNAs (ncRNAs) have emerged as critical components of gene regulatory networks across a plethora of plant species. In particular, the 20–30 nucleotide small ncRNAs (sRNAs) play important roles in mediating both developmental processes and responses to biotic stresses. Based on variation in their biogenesis pathways, a number of different sRNA classes have been identified, and their specific functions have begun to be characterized. Here, we review the current knowledge of the biogenesis of the primary sRNA classes, microRNA (miRNA) and small nuclear RNA (snRNA), and their respective secondary classes, and discuss the roles of sRNAs in plant–pathogen interactions. sRNA mobility between species is also discussed along with potential applications of sRNA–plant–pathogen interactions in crop improvement technologies.

## Introduction

Over the past decade, non-coding RNAs (ncRNAs) have taken the scientific community by storm. Defined as RNAs that are transcribed from DNA but not translated into proteins, ncRNAs have been implicated in a host of different epigenetic regulatory mechanisms and have been shown to exert regulatory effects primarily through complementary base pair matching with messenger RNAs (mRNAs) (Phillips, 2008). However, until recently, ncRNA research has been largely limited by a lack of high-throughput experimental studies, and owing to their small size, ncRNAs within genomic samples were often misidentified as background noise or junk DNA (Grobhans and Filipowicz, 2008; Kawaji and Hayashizaki, 2008; Budak and Zhang, 2017). Thus, the details of the scope and diversity of ncRNAs are only just beginning to emerge following the development of efficient next-generation sequencing (NGS) technologies (Eddy, 2001; Shimoni et al., 2007). When used in combination with homology-based and/or experimental approaches, these NGS-based methods can be particularly worthwhile, because in addition to providing insight into the presence of ncRNAs, they can predict target gene annotations, and thereby reveal genetic circuits that are possibly influenced by ncRNAs (Alptekin et al., 2017).

In the time since their initial discovery, ncRNAs have been segregated based on their biogenesis pathways into a number of different classes, many of which have distinct, yet overlapping, functions (Bonnet et al., 2006). The most diverse range of ncRNAs can be found in the category of small ncRNAs (sRNAs), which are 20–30 nucleotides long and include the widely described small interfering RNAs (siRNAs), microRNAs (miRNAs), and Piwi-interacting RNAs (piRNAs) (Borges and Martienssen, 2015; Budak and Akpinar, 2015). However, numerous smaller classes of sRNAs are beginning to emerge, some of which are conserved only among closely related species. Together, these sRNAs play myriad important roles in plant gene regulation through targeted degradation and/or translational silencing of mRNAs at the post-transcriptional level, collectively termed RNA interference (RNAi) (Pattanayak et al., 2012).

In addition to influencing key developmental processes, RNAi has been shown to have significant impacts on a wide range of stress responses, of which disease resistance is possibly the most vital (Kantar et al., 2011; Budak et al., 2015). Plants are continuously engaged in co-evolutionary battles with invading pathogens, in which the plant and pathogen compete to gain dominance (Dodds and Rathjen, 2010). Furthermore, plant pests and pathogens are considered a major constraint to crop productivity across the globe; in 2016 alone, it was reported that even with current disease management practices in place, 10–16% of global harvests were lost to plant pathogens (Kandan et al., 2016). This included significant losses from many socio-economically crucial crop species, such as wheat (*Triticum aestivum*) and rice (*Oryza sativa*; Fischer et al., 2014; Vary et al., 2015). Moreover, with future climate change currently predicted to favor the invaders, the incidence of disease and infestation is expected to surge (Boyd et al., 2013; Gautam et al., 2013). RNAi could therefore have an important role in the upcoming mêlée, particularly as it has been shown not only to impact resistance gene regulation within a host, but also to target and silence genes within an attacking pathogen or pest (Ghag, 2017; Islam et al., 2018). Observation of this process, termed host-induced gene silencing (HIGS), revealed a plethora of new paths for crop disease management, and highlighted the potential for movement of sRNAs between species (Baulcombe, 2015).

This review article provides an overview of the biogenesis and specific modes of action of four classes of sRNAs: siRNA, miRNA, small nuclear RNA (snRNA, also referred to as U-RNA), small nucleolar RNA (snoRNA), and tasiRNA (*trans*-acting siRNA). A particular focus is placed on the roles of sRNAs in plant–pathogen interactions, including sRNA mobility between species and the applications of HIGS. Finally, future perspectives are explored.

### Biogenesis Differs for Different Classes of sRNAs

In sRNA biogenesis, four key steps are conserved across all classes: (1) as for all coding mRNAs, sRNA transcription is induced by double-stranded RNA (dsRNA), and transcription of the corresponding gene is driven by an RNA polymerase (RNAP); (2) dsRNA is then processed to a length of 18–25 nucleotides; (3) methylation occurs at the 3′ end of the sRNAs; and (4) the sRNAs are incorporated into effector complexes that allow them to recognize, and interact with, their target sites (Voinnet, 2009; Budak and Akpinar, 2015).

For miRNAs, the first step of biogenesis depends on the transcriptional coactivator MEDIATOR, which recognizes the transcription factor-binding sites in miRNA promoters and directs DNA-dependent **RNAP** II (RNAPII) (Chen, 2005; Budak and Akpinar, 2015). Once transcribed, a single-stranded primary miRNA transcript is formed, known as a pri-miRNA, which consists of an imperfect stem-like structure 100–120 nucleotides long. In plants, pri-miRNAs are then processed in the nucleus by the RNase III enzyme Dicer-like protein 1 (DCL1) in combination with a number of different molecules, including a dsRNA-binding protein (HYL1), a nuclear cap-binding complex (CBC), and a C2H2-type zinc finger (SE) (Fang and Spector, 2007; Zhu, 2008; Axtell and Meyers, 2018). This DCL1 complex first cleaves approximately 15 nucleotides from the base of the stem to form a precursor miRNA (pre-miRNA) approximately 70 nucleotides long with **two to three** nucleotide overhangs. A second cleavage then occurs 20–24 nucleotides from the first cleavage site, releasing the miRNA/miRNA^∗^ (guide strand/passenger strand) duplex from the stem (Rogers and Chen, 2012, 2013). This duplex is protected from degradation by the small RNA degrading nuclease (SDN) class of endonucleases via methylation by HEN1, before the miRNA guide strand is incorporated into an Argonaut (AGO)-containing RNA-induced silencing complex (RISC) (Voinnet, 2009; Axtell et al., 2011). Previously it was assumed that exportation into the cytoplasm could occur either before or after RISC assembly (Achkar et al., 2016). However, recent research by Bologna et al. (2018) strongly supports the hypothesis that movement into the cytoplasm occurs post-incorporation into RISCs.

To add another layer of complexity to the process of sRNA biogenesis, tasiRNAs are initially formed from primary tasiRNAs (pri-tasiRNA) via miRNA-guided cleavage, which creates *TAS* transcripts (Chen et al., 2010). Three miRNAs (miR173, miR390, and miR828) specifically function as processing guides for primary transcripts to form the *TAS1* and *TAS2* (miR173), *TAS3* (miR390), and *TAS4* (miR828) families, respectively (Howell et al., 2007). These precursor transcripts are then stabilized by *SUPPRESSOR OF GENE SILENCING 3* (SGS3), which facilitates dsRNA formation by RNAPII. The dsRNAs are then processed by DCL4, yielding phased siRNAs that are 21 nucleotides long, which, like miRNAs, are methylated by HEN1 and loaded into AGO-containing RISCs (Brodersen and Voinnet, 2006). tasiRNAs are also similar to miRNAs in function, as they guide the cleavage and degradation of mRNAs. Together, miRNAs and tasiRNAs regulate numerous biological processes, of which auxin production is one of the most researched (Marin et al., 2010).

However, in general, biogenesis of siRNAs is much more diverse than miRNAs, and can differ greatly between siRNA classes. Initially, most siRNAs are conceived from pre-formed dsRNAs. This includes, but is not limited to, those transcribed by RNA-dependent RNAPs (RDRs) (Khraiwesh et al., 2012). siRNA formation can also utilize a number of different DCL proteins (DCL1–4), opposed to just one, which cleave the dsRNAs to form the different classes of siRNA, defined by size (21–24 nucleotides). However, some similarities have been observed as like miRNAs, siRNAs are also loaded into AGO-containing RISCs for targeted post-transcriptional regulation (Matzke and Mosher, 2014; Martínez de Alba et al., 2015).

In comparison, little is known about the specifics of snRNA biogenesis in plants, as most research has been conducted in mammalian cells and the process varies depending on the specific snRNA being transcribed (Will and Lührmann, 2011; Ohtani, 2018). Generally, like miRNAs, snRNAs are transcribed by RDR2, but are primarily confined to the nucleus (Vaucheret, 2006). During their biogenesis, snRNAs briefly move into the cytoplasm of the cell before returning to the nucleus. Following transcription, snRNAs form protein complexes with specific snRNA-associated proteins to shape small nuclear ribonucleoproteins (snRNPs) (Matera and Wang, 2014). These snRNPs have corresponding functional spliceosomal roles in pre-mRNA splicing (Hanley and Schuler, 1991).

Another major group of snRNAs is involved in regulating RNA metabolism. Within this group, one sub-class, known as scaRNAs (small Cajal body-specific RNAs), directs a group of snRNPs known as the scaRNPs (small Cajal body-specific RNPs), which have been implicated in pre-ribosomal RNA (pre-rRNA) modification and processing in the Cajal bodies. A second sub-class, the snoRNAs, is mainly located in the nucleolus and form snoRNPs, which are involved in regulating and modifying other spliceosomal snRNAs (Chen et al., 2003; Kim et al., 2010; Bassett, 2012; Ohtani, 2018). The current consensus is that the post-transcriptional modifications made to snRNAs by snoRNAs and scaRNAs convey their binding affinity to pre-mRNAs, and that this process is conserved among most eukaryotes, including plant species. However, little research has been conducted in plants to confirm this (Lorković and Barta, 2008; Ohtani, 2018). snRNAs play a pivotal role in mRNA metabolism, as the removal of ncRNA (introns) from pre-mRNA is crucial to the formation of mature mRNAs, although little research has examined their role in plant–pathogen interactions (Lorković et al., 2000; Monaghan et al., 2009).

### sRNAs Regulate Plant–Pathogen Interactions

Plant immune systems consist mainly of two different modes of defense, pathogen-associated molecular pattern (PAMP)-triggered immunity (PTI) and effector-triggered immunity (ETI) (Boller and He, 2009; Fei et al., 2016). In most cases, PTI acts as the first line of defense against invading pathogens, with pattern recognition receptors (PRR) on the plant cell surface recognizing PAMPs present on the pathogen, such as flg22 found on *Pseudomonas aeruginosa* (Jones and Dangl, 2006). This pathway can initiate a number of different responses, including stomatal closure and activation of reactive oxygen species (Stael et al., 2015). However, the bulk of plant innate immunity is managed by ETI, which recognizes effector molecules secreted by pathogens. Most, if not all, immune responses elicited are mediated by nucleotide-binding leucine-rich repeat (NB-LRR) proteins, also known as NB-LRR or NLR proteins, encoded by *NLR* genes, which are commonly referred to as resistance genes (*R*-genes) (Ellis et al., 2000). It is routinely acknowledged that RNAi plays a large role in *R*-gene regulation in plants. As *R*-gene activation is required only in the presence of an invading pathogen, regulation is the key for efficient management of plant resources, allowing sufficient energy to be directed toward relevant processes at any given time (Zhai et al., 2011). Furthermore, unregulated expression of *R*-genes has been known to trigger autoimmunity and further inhibit plant growth, showcasing the impact of inadequate energy trade-off (Li et al., 2012). In particular, miRNAs, and the subsequent tasiRNAs, have major impacts on *R*-gene regulation, with research predicting *R*-gene targets for miRNAs in numerous plant species, including wheat, tobacco (*Nicotiana tabacum*), and cotton (*Gossypium hirsutum*; Gupta et al., 2012; Li et al., 2012; Yin et al., 2012; Budak et al., 2015). An example of this is the miRNA family miR482, which is known to target a class of NB-LRR proteins in a number of different species (Shivaprasad et al., 2012). In potato (*Solanum tuberosum*) specifically, members of the miR482 family have been shown to be downregulated upon infection with the fungal pathogen *Verticillium dahliae* (Yang et al., 2013). Furthermore, mutant plants that overexpress miR482e also exhibit hypersensitivity to V. dahliae due to production of miR482-derived tasiRNAs (Yang et al., 2015).

Small RNAs have also been shown to indirectly regulate *R*-gene expression by targeting genes related to co-expression of *R*-genes, and thereby contributing to negative-feedback loops (Yang and Huang, 2014). For instance, mutant *Arabidopsis* plants defective in small RNA biogenesis (such as *ago1-36* and *dcl4-1*) have elevated levels of SNC1 transcript, whose overexpression triggers downregulation of *R*-genes in the RPP5 locus via co-suppression (Yi and Richards, 2007). This suggests that *SNC1* is most likely repressed by a small RNA pathway during pathogen infection, which enables expression of the relevant *R*-genes (Yi and Richards, 2007).

Furthermore, targeting of *NB-LRRs* by miRNAs has been shown to trigger production of phased secondary siRNAs (phasiRNAs) from their target mRNA cleavage site (Allen et al., 2005). These are mainly formed by miRNAs 22 nucleotides in length, of which one of the most frequent is miR393, which is involved in auxin signaling and is commonly conserved across species. The biogenesis of phasiRNAs is similar to that of tasiRNAs, and phasiRNAs also have the ability to target transcripts for degradation, often continuing to target the same NB-LRR family as the initial miRNA. However, little else is known about their function (Zhai et al., 2011; Fei et al., 2016).

Besides tasiRNAs and phasiRNAs, a number of other siRNAs also hold the potential to play important roles in disease resistance. Some of these roles involve direct interactions with the pathogen and have already been used to show resistant phenotypes in mutant plants (Casacuberta et al., 2015; Machado et al., 2017). An example of this was highlighted by Zrachya et al. (2007), who used siRNAs derived from an intron-hairpin RNA (ihpRNA) to target the coat protein (CP) of the *Tomato yellow leaf curl virus* (TYLCV) in tobacco and induce resistance. Additionally, technology has been developed to help analyze the overall expression patterns of sRNAs in the genome, known as sRNA high-throughput sequencing. As demonstrated by Guo et al. (2015) with transgenic anti-*Rice stripe virus* (RSV) rice plants, this technology can help pinpoint which sRNAs play dominant roles when a host is infected, along with a multitude of other stress-inducing conditions (Luan et al., 2015; Mishra et al., 2016; Li et al., 2017).

Both in combination and individually, miRNAs and siRNAs offer myriad opportunities to improve disease-resistant traits in crops. Knowledge of sRNAs, if successfully amalgamated with innovative gene editing technologies such as CRISPR, has the potential to create a new path for crop breeding, allowing targeted editing at the post-transcriptional level (Liu and Chen, 2010; Tang and Chu, 2017). This would not only expand knowledge of gene regulation and function considerably, but also pave the way for efficient targeted modifications of the epigenome (Basak and Nithin, 2015; Zhou et al., 2017). However, the application of these sRNAs in crop improvement has yet to be sufficiently explored, as reliable high-throughput data on miRNA-target pairs in many crop species are unavailable, and only a few small-scale studies have been completed (Jacobs et al., 2015; Zhao et al., 2016; Zhou et al., 2017). This does, however, highlight a possible route for future exploration (Tang and Chu, 2017).

As snRNAs are primarily involved in splicing activities in the nucleus, little research has attempted to solidify their associations with plant–pathogen interactions. However, Monaghan et al. (2009) reported a suspected interaction of snRNP proteins with the MOS4-associated complex (MAC), which is essential for suppression of *SNC1*-mediated autoimmunity. Their study suggested that snRNPs were required to initiate basal and *R*-protein-mediated resistance in *Arabidopsis*. However, this reaction has not been quantified, and other associations of sRNA with plant–pathogen interactions have not been identified, leaving an open niche for future research.

### sRNA Mobility Regulates Plant–Pathogen Interactions

Another mode by which sRNAs influence plant–pathogen interactions involves the movement of sRNAs both within and between organisms. Intercellular movement of sRNAs is still relatively unexplored, as RNAs were previously assumed to remain within a single cell due to their vulnerability to nuclease degradation (Melnyk et al., 2011). However, this view began to change after short-range cell-to-cell movement was first observed in plants in 1997 (Palauqui et al., 1997; Voinnet and Baulcombe, 1997). Following this, over the past 20 years, both intra- and inter-species long-range mobility has now been observed, including not only plant-to-pathogen transportation but also the reverse process, where pathogen sRNAs move into plant cells (Knip et al., 2014). Interestingly, plant sRNAs can also be transported through a number of different plant species via a parasitic plant intermediate that infects them all, allowing sRNA mobility between plant species (Melnyk et al., 2011; Dunoyer et al., 2013).

Within a plant, sRNAs are typically thought to use a symplastic form of movement to achieve cell-to-cell mobility through the plasmodesmata as many sRNAs have already been shown to be present within phloem sap (Zhang et al., 2009). This allows sRNAs to move from photosynthetic tissues to growing points and sinks (Himber et al., 2003; Dunoyer et al., 2013; Lewsey et al., 2016). However, the specifics of this mechanism, and the exact functions of the sRNAs in the phloem are still very vague. Ham et al. (2014) showed that in pumpkin (*Cucurbita maxima*) a PHLOEM SMALL-RNA BINDING PROTEIN 1 (PSRP1) acts as a major component in the formation of a sRNA ribonucleoprotein complex (sRNPC), which allows movement of sRNAs into sink organs. In speculation, this mechanism could represent a method of RNA silencing-mediated resistance, as it gives a plant the ability to recover from viral infection. This may result in the upper regions of a plant exhibiting secondary resistance to a viral infection, appearing phenotypically disease free compared to the infected lower regions (Molnar et al., 2011; Pyott and Molnar, 2015). This is an interesting prospect, as it in essence implies the existence of an adaptive form of immunity that would require the sRNAs to be initially present only in some cells, not all. However, these changes do not appear to be hereditary and are initiated only in the infected plant, suggesting that if they arose in crops, a loss of yield would first need to occur before the crop could gain immunity (Brosnan et al., 2007; Melnyk et al., 2011).

The ability of sRNAs to move within the plasmodesmata is also thought to be an important aspect of initial host response to disease, as some infectious agents, such as phytoplasmas, are restricted in movement and are commonly localized to the sieve elements of the phloem tissues (Bertaccini and Duduk, 2009). It is therefore assumed that the host response must also occur within the phloem. An example of this was identified in mulberry (*Morus* sp.) which, when investigated, highlighted 43 miRNAs, 13 of which were novel, that were differentially expressed upon infection with yellow dwarf disease phytoplasmas. In particular, mul-miR482a-5p was shown to hold the ability to move from scions to rootstock, and was upregulated upon infection (Gai et al., 2014, 2018).

**FIGURE 1:**
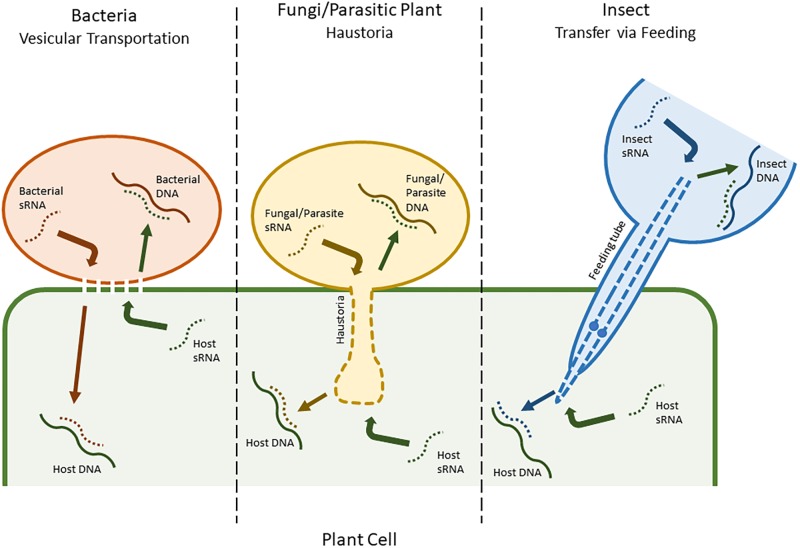
A putative representation of different modes of small RNA (sRNA) mobility, including between plant cells and bacteria (vesicular transportation), plant cells and fungi/parasitic plants (haustoria), and plant cells and insect herbivores (feeding tube).

Many plant sRNA silencing machineries also have the ability to recognize and process viral dsRNAs, and consequently create virus-derived siRNAs that combine with RISCs to silence viral genes in a natural antiviral process (Duan et al., 2012). This has also been adapted as a molecular tool, known as virus-induced gene silencing (VIGS). In retaliation, viruses have evolved mechanisms, such as viral suppressors of RNA silencing (VSR), that suppress host genes involved in sRNA production (Dunoyer and Voinnet, 2005). These suppressor proteins may also inadvertently silence other host regulatory sRNAs (Dunoyer et al., 2004; Moissiard and Voinnet, 2004). However, more complex mechanisms and in-depth manipulations have also been implicated, and many other types of pathogens have recently been shown to have evolved similar means of reprisal (Chapman et al., 2004; Yang and Huang, 2014). An example of this, detailed by Weiberg et al. (2013), is used by the fungal pathogen *Botrytis cinerea*, which releases sRNAs into plant cells with the ability to hijack host AGO proteins and target genes related to immunity. Some *Phytophthora* species have also been shown to produce effector molecules that are able to interfere with host sRNA silencing (Ye and Ma, 2016). Similarly, plants have a comparable system for battling other pathogens, known as HIGS (Ghag, 2017). This also facilitates plant sRNAs in targeting genes in an invading pathogen’s genome. Unlike with viruses, where the viral genome is freely accessible in the host cell, for HIGS to be successful, sRNAs need to be transferred between species (Knip et al., 2014). The specific mechanisms driving this process are largely under debate, with the mechanisms in bacteria being the most disputed. Different species likely use a variety of mechanisms. Two of the most commonly proposed methods are transfer via vesicular transportation in bacteria and via haustoria in fungi (**Figure 1**; An et al., 2006; Nowara et al., 2010; Zhang et al., 2012; Hua et al., 2018).

Mobility has also been observed between larger organisms, including both insect pests and other plant species (Mao et al., 2007; Knip et al., 2014). Tomilov et al. (2008) reported that sRNAs had the ability to move between parasitic plants, such as *Triphysaria versicolor* and *Orobanche aegyptiaca*, and their corresponding hosts in a similar fashion to fungal pathogens, via the haustoria (Westwood et al., 2009). For insect pests, the mode of mobility is slightly different, and usually consists of sRNAs being taken up during feeding (**Figure 1**). However, although this mechanism is efficient in sucking insects and nematodes, some species of insect, including phloem sap-feeding hemipterans such as the global crop pest *Bemisia tabaci* (Luo et al., 2017), exhibit a low RNAi efficiency due to adaptations that allow them to degrade dsRNA before it can have an effect (Whyard et al., 2009; Xue et al., 2012; Scott et al., 2013). Nonetheless, insect RNAi has already been tested as a tool for crop improvement with varying levels of success in crop pests such as *Leptinotarsa decemlineata* (Colorado potato beetle), *Helicoverpa armigera* (cotton bollworm), and *Diabrotica virgifera* (Western corn rootworm) (Baum et al., 2007; Mao et al., 2007; Zhang et al., 2015).

Furthermore, both VIGS and HIGS have been used as innovative molecular techniques to manage plant pathogens. These have been evaluated in both dicot and monocot plants, and work through introducing engineered sRNAs via *Agrobacterium tumefaciens* (Holzberg et al., 2002; Yin and Hulbert, 2015). Following this, RNAi methods have been successfully applied to induce sRNA silencing of target genes in a number of different pathogens, including *Blumeria graminis* and *Puccinia striiformis* f. sp. *tritici*, in a fast, efficient manner. Post-infection, only 3–4 weeks are needed to attain resistance, and one of the greatest benefits of using such methods is that, in principle, the only knowledge needed to target a pathogen or insect gene is the gene sequence itself (Burch-Smith et al., 2004; Nunes and Dean, 2012).

However, the HIGS and VIGS are both limited in their applications, as they require efficient plant transformation protocols, which are not always available for complex crop species such as wheat. There is also some uncertainty surrounding the resultants plants status as a genetically modified organism (GMO), eliciting both public and regulatory concerns. To address these issues, alternative technologies have also been trialed to explore other possible pathways for induction of RNAi (Robinson et al., 2014). One such technology involves the exogenous application of dsRNA to host plants, which induces temporary RNAi-mediated defense. This method is in very early stages, and most research is currently focused on optimizing the delivery method to increase the length of time resistance is induced for (Mitter et al., 2017b). To date, the most effective manner of delivery is combining naked dsRNA with layered double hydroxide (LDH) nanosheets, also known as BioClay, which are then sprayed onto plants. In tobacco plants, this has been shown to induce resistance for up to 20 days post-spraying (Jiang et al., 2014; Mitter et al., 2017a).

All three of the methods above, HIGS, VIGS, and naked dsRNA application, have important implications for crop breeding efforts, as they not only highlight novel techniques for potentially improving crop resistance to pathogens, but also help to shed light on possible mechanisms and genes that may already be at play, and efficient ways of investigating them (Lu et al., 2003; Burch-Smith et al., 2004; Nowara et al., 2010). However, these techniques are still under development, and need to be developed further before the full scope of their applications can be appreciated (Kusaba, 2004; Perrimon et al., 2010; Koch and Kogel, 2014).

## Author Contributions

EB wrote the paper. HB conceived the idea and edited the paper.

## Conflict of Interest Statement

The authors declare that the research was conducted in the absence of any commercial or financial relationships that could be construed as a potential conflict of interest.
